# Cryo-EM structures of peripherin-2 and ROM1 suggest multiple roles in photoreceptor membrane morphogenesis

**DOI:** 10.1126/sciadv.add3677

**Published:** 2022-11-09

**Authors:** Dounia El Mazouni, Piet Gros

**Affiliations:** Structural Biochemistry, Bijvoet Centre for Biomolecular Research, Department of Chemistry, Faculty of Science, Utrecht University, Netherlands.

## Abstract

Mammalian peripherin-2 (PRPH2) and rod outer segment membrane protein 1 (ROM1) are retina-specific tetraspanins that partake in the constant renewal of stacked membrane discs of photoreceptor cells that enable vision. Here, we present single-particle cryo–electron microscopy structures of solubilized PRPH2-ROM1 heterodimers and higher-order oligomers. High-risk PRPH2 and ROM1 mutations causing blindness map to the protein-dimer interface. Cysteine bridges connect dimers forming positive-curved oligomers, whereas negative-curved oligomers were observed occasionally. Hexamers and octamers exhibit a secondary micelle that envelopes four carboxyl-terminal helices, supporting a potential role in membrane remodeling. Together, the data indicate multiple structures for PRPH2-ROM1 in creating and maintaining compartmentalization of photoreceptor cells.

## Teaser

PRPH2-ROM1 heterodimeric and oligomeric structures underlie the membrane shaping of the photoreceptor cell outer segments.

## INTRODUCTION

Mammalian vision relies on continuous renewal of hundreds of tightly packed membrane discs that form the photosensory organelles, called outer segments (OS), in rod and cone photoreceptor cells (*1*, *2*). Key players in the initialization, maturation, and stability of the OS morphology are peripherin-2 (PRPH2) and its homolog retinal outer segment protein 1 (ROM1) (*3*, *4*). In a multifaceted and dynamic process, membrane protrusions are formed by evagination of the ciliary plasma membrane, followed by membrane expansion into lamellae (*5*–*8*). In rod cells, a process of rim advance closes the edges of lamellae and creates internalized discs (*5*, *9*). Dependent on species, discs may be split by one or more incisures, forming axial tunnels used for the diffusion of soluble molecules required for phototransduction (*9*). A broad range of genetic mutations disrupt the OS morphology, leading to blindness (*10*). Many of those mutations are located in the PRPH2 gene and are linked to various inherited human retinal diseases (*11*). In mice, two PRPH2 functioning alleles are required for proper development and function of OS (*3*). Lack of PRPH2 inhibits rod OS formation and leads to an extensive amount of ectosome release from the photoreceptor cilium (*12*, *13*). In cone cells, the OS undergoes severe disorganization (*14*). In transgenic frogs, PRPH2 mutants causes abnormal incisures (*15*). Few disease-related mutations are associated to ROM1. However, heterozygous ROM1 mice exhibit enlarged discs, indicating a role for ROM1 in regulating disc morphogenesis (*16*, *17*).

PRPH2 and ROM1 are retina-specific members of the tetraspanin family. The members of this family, 33 in humans, are known as membrane organizers and are involved in formation of extracellular vesicles (*18*). They are characterized by four transmembrane (4-TM) helices, one small extracellular loop (EC1) and one large extracellular loop (EC2). PRPH2 and ROM1 EC2 carry an odd number of cysteines that allows homo- and hetero-oligomerization via intermolecular disulfide bond formation, a prerequisite for OS morphogenesis (*10*). PRPH2 alone and in combination with ROM1 forms large oligomers, whereas ROM1 on its own forms complexes of intermediate size (*15*). Recent cryo–electron tomography (cryo-ET) performed on mouse rod OS indicated that PRPH2-ROM1 heterodimer is the minimal unit of oligomerization and revealed three dimers arranged in a sharp negative curve stabilizing disc rims, reinforcing PRPH2-ROM1’s role in OS structural integrity (*19*). However, the molecular basis and mechanisms underlying OS morphogenesis remain largely unresolved.

## RESULTS AND DISCUSSION

**Fig. 1.**
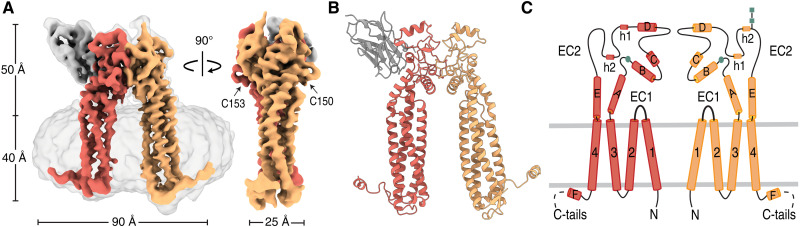
Cryo-EM structure of PRPH2-ROM1 heterodimer. (**A**) Overall cryo-EM map with ROM1 and PRPH2 is colored red and orange, respectively. Detergent micelle density is transparently visible (left). Nanobody19 is shown in gray. (**B**) Ribbon representation of PRPH2-ROM1 dimer in the same orientation and colors as the map in (A). (**C**) Topology diagram of PRPH2-ROM1 showing secondary structure elements, same colors as in (A). Free cysteines in the AB loops and *N*-acetylglucosamine shown as green squares and sticks, respectively.

Here, we coexpressed human PRPH2 and ROM1 transiently in human embryonic kidney (HEK) 293T cells and solubilized them in detergent *n*-dodecyl-β-d-maltoside (DDM) and cholesteryl hemisuccinate (CHS). Size-exclusion chromatography (SEC) yielded a broad set of overlapping peaks, indicating a range of oligomeric species (fig. S1A). Addition of dithiothreitol reduced all forms to the smallest molecular weight species confirming that oligomerization is dependent on disulfide bond formation (fig. S1E). Mutation of PRPH2 C150S resulted in one homogeneous peak (fig. S1F), whereas mutation of ROM1 C153S yielded two peaks, consistent with a PRPH2 dimer and monomer-dimer distribution of ROM1 (fig. S1G). For single-particle cryo–electron microscopy (cryo-EM) structure determination of PRPH2-ROM1, we expressed and purified PRPH2-ROM1 with mutation C150S in PRPH2 to avoid oligomerization and added a nanobody to increase cryo-EM particle size. For this, we used Nanobody19, which is described to bind bovine PRPH2-ROM1 complexes (*20*), thus taking advantage of the high sequence homology between bovine and human EC2s of these proteins (fig. S2). Cryo-EM micrographs showed nicely distributed particles and two-dimensional (2D) class average revealed one nanobody bound to the extracellular part of the micelle-embedded PRPH2^C150S^-ROM1 complex (fig. S3). The resulting cryo-EM density map revealed a heterodimer of PRPH2^C150S^-ROM1 at a resolution of 3.7 Å (Fig. 1A and fig. S4).

The well-resolved density map allowed us to build nearly complete atomic models for both PRPH2 and ROM1 and Nanobody19 that exclusively interacts with the extracellular domain of ROM1 (Fig. 1B and fig. S2). The short EC1 regions of both PRPH2 and ROM1 form tight turns between the extended TM helices 1 and 2 (fig. S4). The PRPH2 and ROM1 display classical tetraspanin EC2 folds, with helices A to E, but are extended (compared to other tetraspanins) by more than 80 residues forming loops and two short helical turns, h1 and h2 (Fig. 1C). Three disulfide bonds, Cys^217^-Cys^225^, Cys^169^-Cys^216^, and Cys^168^-Cys^253^ in ROM1 and Cys^165^-Cys^250^, Cys^166^-Cys^213^, and Cys^214^-Cys^222^ in PRPH2, stabilize the EC2 regions (fig. S5). The free cysteines, PRPH2 Cys^150^ and ROM1 Cys^153^, are positioned in the AB loop and are freely accessible for potential oligomerization (Fig. 1A). The C-terminal extensions form amphipathic helices (*21*, *22*), residues 312 to 321 in PRPH2 and residues 315 to 322 in ROM1, which are positioned at the micelle-water interphase. However, the C-terminal tails of the PRPH2-ROM1 heterodimer could not be modeled fully, with residues 322 to 346 of PRPH2 and residues 323 to 351 of ROM1 being too flexible for structure determination. Density for a *N*-acetylglucosamine moiety was observed in the cryo-EM map on top of PRPH2 molecule indicating glycosylation of residue Asn^229^, while the map suggests possible glycosylation at Asn^53^, although this density was less resolved.

**Fig. 2.**
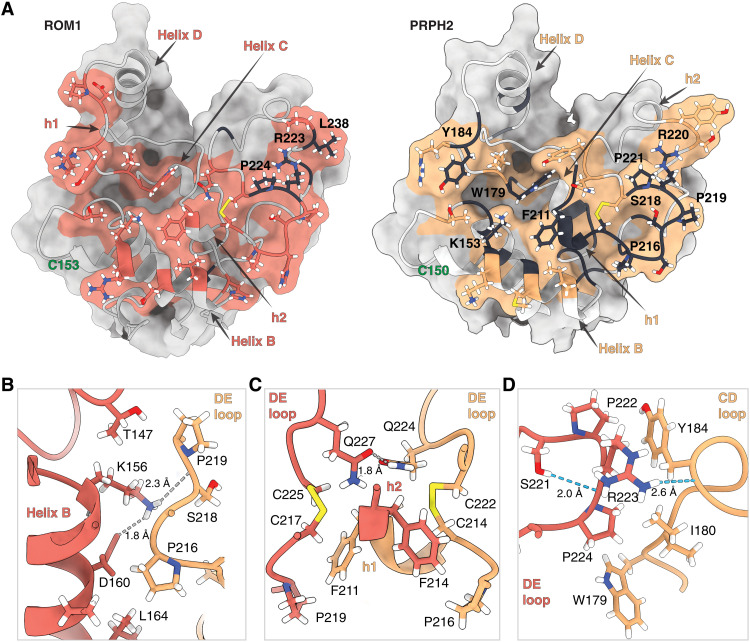
PRPH2-ROM1 heterodimer interface and structural interpretation of degenerative retinal diseases mutations. (**A**) Open-book surface representation of the PRPH2-ROM1 interface is colored orange and red, respectively. High-risk human mutations are mapped onto the structure in black. Cysteine residues that permit oligomerization via intermolecular disulfide bond formation, PRPH2 C150 and ROM1 C153, are shown in green. (**B** to **D**) Zoomed-in presentations of PRPH2-ROM1 interactions.

PRPH2 and ROM1 dimerize through a head-to-head assembly of their respective ectodomains yielding a molar-like appearance (Fig. 2A). The interface is formed by the respective EC2 regions and buries a total surface area of 1195 Å^2^. The overall molecular architecture creates a crevice with helices D at the top and helices B on the bottom. Helix B of both molecules are facing each other, held by a pair of hydrogen bonds between PRPH2 Lys^153^ and ROM1 Ser^221^ and PRPH2 Ser^218^ and ROM1 Lys^156^. Hydrophobic interactions between the side chains of PRPH2 Met^158^, His^167^, and Ile^161^ and ROM1 Pro^219^, His^167^, and Leu^164^ contribute to the dimer interface (Fig. 2B). PRPH2 h1 and ROM1 h2 are localized ~5 Å above their respective B helices, and both accommodate the hydrophobic PRPH2 Phe^211^ and ROM1 Phe^214^ facing ROM1 Pro^219^ and PRPH2 Pro^216^, respectively (Fig. 2C). A hydrogen bond between PRPH2 Gln^224^ and the carbonyl oxygen of ROM1 Gln^227^ seals the heterodimer at the center of the interface (Fig. 2C). On the peripheries of the dimer crevice, we observed van der Waals contacts between the side chains of PRPH2 Tyr^234^ and ROM1 Asp^189^ and Pro^190^ and another hydrophobic patch between PRPH2 Pro^221^ and ROM1 Trp^221^ and Val^183^ (Fig. 2D). A hydrogen bond links PRPH2 Arg^220^ and ROM1 Tyr^187^. On the opposite peripheral side, we also observed a cluster of hydrophobic residues, PRPH2 Ile^180^, Leu^185^, and ROM1 Pro^224^, Leu^238^, and a final hydrogen bond between PRPH2 Tyr^184^ and ROM1 Arg^223^, further stabilizing the heterodimer interface (Fig. 2E).

In humans, 166 disease-causing mutations have been identified in PRPH2 and 15 in ROM1 (table S1) [Human Gene Mutations Database (*23*)]. These mutations relate to a broad phenotypic spectrum: 83 mutations are associated with retinitis pigmentosa phenotype, 33 mutations for pattern dystrophy, 24 mutations for macular dystrophy, 19 mutations for Stargardt disease, and 10 mutations in PRPH2 for macular and cone/cone-rod dystrophy. Of these mutations, 127 PRPH2 mutations are located in the EC2 domain (Fig. 2A and table S1). All 47 high- to very high–risk mutations in PRPH2 map onto dimer interface, except for Arg^195^ located in the helix D of the periphery of the interface crevice, underscoring the importance of hetero- or homodimerization in PRPH2 function. The remaining 80 mutations in PRPH2 EC2 are scattered throughout and are mostly buried, suggesting that these mutations are deleterious by destabilizing the molecule. Seven mutations in ROM1 are in the EC2 domain, with Pro^224^, Arg^223^, and Leu^238^ mapped onto the periphery of the PRPH2-ROM1 interface (Fig. 2D), indicating the importance for this region for ROM1 activity. Consistently, alignment of 100 PRPH2 and ROM1 homologs by ConSurf (*24*) showed high conservation for the PRPH2-ROM1 interface and the AB loops that carry the free cysteines, which emphasizes the critical role for noncovalent PRPH2-ROM1 dimerization and disulfide bond–driven oligomerization (fig. S7).

In the heterodimer, PRPH2 and ROM1 form two separate bundles of 4-TM helices (Fig. 1B). The 4-TM bundles of PRPH2 and ROM1 are tightly packed and do not exhibit central cavities, consistent with predictions by AlphaFold (*25*) (fig. S6). In contrast, known structures of other (noncomplexed) tetraspanins display cone-shaped arrangements of their 4-TM helices (*26*–*28*). However, tetraspanin CD81 in complex with CD19 displays a cylindrical 4-TM arrangement (*29*), indicating a conformational change upon complex formation. Potentially, prearranged cylindrical 4-TM structures of PRPH2 and ROM1 may be indicative of a high propensity for complex formation; in agreement with this, we observed only dimers of PRPH2 and mostly dimers of ROM1 (fig. S1G). Superimposition of AlphaFold structure predictions of all individual 33 human tetraspanins classified them into three structural groups: one group with a cylindrical shaped 4-TM helices (fig. S8E) and two groups displaying a narrow and a wide cone (fig. S8, C and D) (*25*). The cylindrical bundle group encompasses three other tetraspanins, uroplakin 1a, uroplakin 1b, and TSPAN32, in addition to PRPH2 and ROM1 (fig. S8E). Uroplakin 1a and 1b are uniquely expressed in umbrella cells that are responsible for expansion and contraction of the bladder (*30*). Similar to PRPH2 and ROM1, they are required for storage of large intracellular membrane structures (*30*, *31*), indicating that they may potentially execute their function through related mechanisms.

**Fig. 3.**
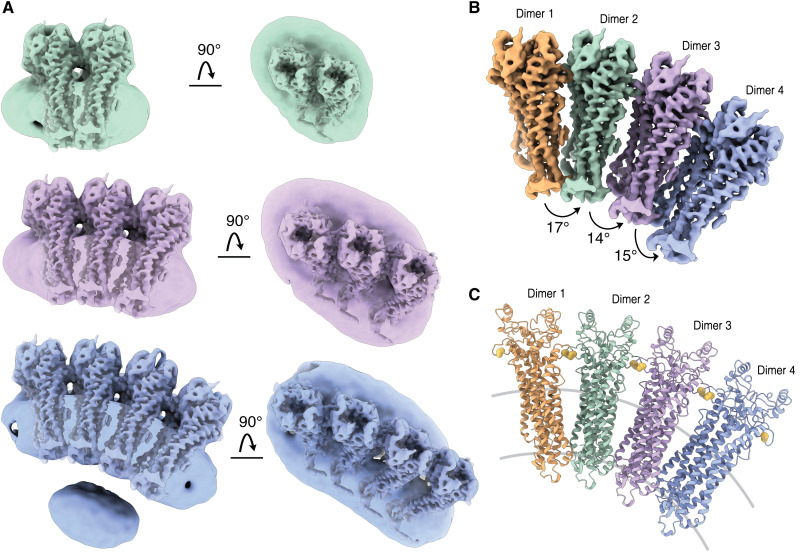
Cryo-EM structures of PRPH2-ROM1 oligomers. (**A**) Cryo-EM map of PRPH2-ROM1 tetramer (green), hexamer (violet), and octamer (blue). Side view and top view are shown. High-resolution dimers fitted into corresponding density are transparently visible. (**B**) Cryo-EM model of PRPH2-ROM1 octamer with each dimer colored separately. (**C**) Ribbon representation of PRPH2-ROM1 octamer in the same orientation and colors as the map in (B). Intermolecular cysteines connecting dimers are shown in yellow.

**Fig. 4.**
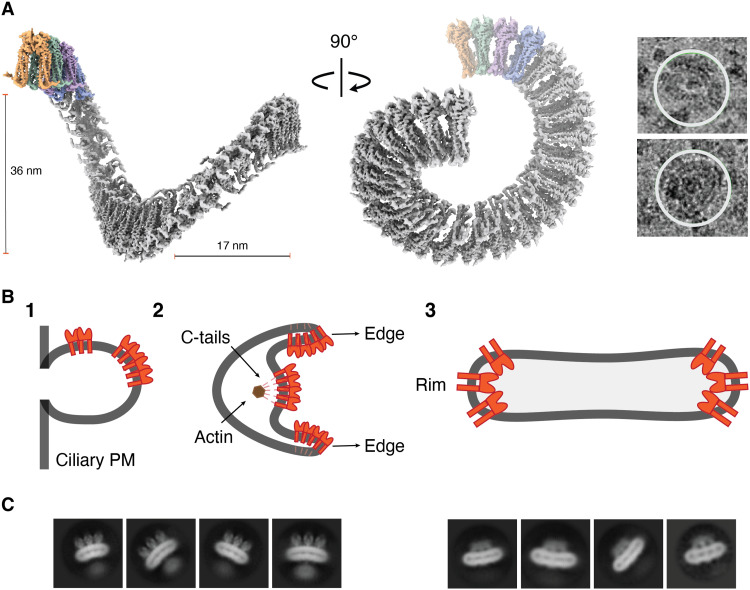
Disc morphogenesis model. (**A**) Twisted ribbon model of 50-nucleotide oligomer PRPH2-ROM1. Particles present in micrographs highlighted using a white 460-Å circle. (**B**) Schematic representation of steps in disc morphogenesis based on Steinberg *et al.* (*5*) and a model depicting how PRPH2 and ROM1 play a role in this process: (1) membrane evagination of the ciliary plasma membrane (PM), (2) bilateral membrane expansion and enclosure, and (3) mature disc with PRPH2-ROM1 scaffold at disc rims according to Pöge *et al.* (*19*). PRPH2-ROM1 dimers are schematically indicated in red, and membrane is indicated in dark gray. (**C**) 2D classes of PRPH2-ROM1 hexamers showing negative curvature (right) and positive curvature (left). Negative curvature reflects a scaffold of three noncovalent dimers as observed in mature disc rim (*19*). Positive curvature complexes reveal dimers covalently linked through disulfide bonds exhibiting a secondary micelle that we hypothesize surrenders the C-terminal tails and may support the first steps of disc morphogenesis.

Negative-stained EM micrographs of solubilized PRPH2-ROM1 complexes showed hetero-oligomeric particles varying in curvature and size (ranging from 10 to 123 nm length; fig. S9) resembling PRPH2-ROM1 tubules reported previously (*15*). Single-particle averaging of cryo-EM data of oligomeric complexes, using the “build and retrieve” methodology (*32*), yielded density maps of PRPH2-ROM1 tetramers, hexamers, and octamers up to resolutions of ~7 Å (Fig. 3 and fig. S10). Pseudo-atomic resolution models were obtained by docking the high-resolution heterodimer map into the cryo-EM density map of each oligomer structure (Fig. 3A). These models revealed a staggered alignment of PRPH2-ROM1 dimers into positively curved oligomers that are held together exclusively via intermolecular disulfide bridges (fig. S11). However, the alignment appears irregular because we observed a variation of 14° to 17° angle between neighboring dimers (Fig. 3B). This reflects potential inhomogeneity in PRPH2 and ROM1 composition within these oligomers. In addition, approximately 20% of tetrameric particles display an alternative form with a contact interface between the EC2 heads (fig. S12A) possibly corresponding to interactions observed in oxidized tetramers (*15*). Furthermore, we observed occasionally hexamers with negative curvature (Fig. 4C). This packing of EC2 heads likely does not involve the intermolecular disulfide bridge and is reminiscent (although with relaxed curvature) of that of the highly negatively curved arrangement within disc rims observed by cryo-ET (*19*).

2D and 3D class averages of PRPH2-ROM1 hexamers and higher-order oligomers revealed an extraglobular density located at the cytosolic side (fig. S13). This density is ~45 Å in diameter and appears connected to four C-terminal helices derived from three protein dimers, with a central dimer providing two termini and opposing neighboring dimers providing each one terminus (fig. S13A). The size of the globular density matches with an extension of ca. 50 amino acid–long helices that are embedded in a micelle (fig. S13B). This is in marked agreement with an AlphaFold prediction of a C-terminal helix formed by PRPH2 residues 296 to 346 (fig. S13C). In the PHPH2-ROM1 heterodimer, we observed an amphipathic C-terminal helix at the water-micelle interface formed by PRPH2 residues 312 to 328, thus suggesting a conformational rearrangement of C-termini upon oligomerization. Concordantly, an enhancing α-helical content of C termini (upon changing membrane conditions) has been suggested to increase the presence of lipids (*33*). Functionally, PRPH2 C terminus promotes OS targeting and suppresses ectosome retention in OS morphogenesis, which constitute the first steps of membrane evagination during disc formation (*13*, *34*–*36*). Our data suggest that C-terminal tails may undergo changes upon oligomerization and associate giving rise to a second hydrophobic milieu, which is possibly important to support steps of plasma membrane growth and remodeling that contribute to disc morphogenesis (Fig 4).

The EM micrographs also showed the occurrence of large multimers (Fig. 4A). To model these multimers, we extended the pseudo-atomic PRPH2-ROM1 octamer model into 25 heterodimers, yielding a twisted ribbon–like structure with projections that mimic the particles present in the cryo-EM micrographs (Fig. 4A). Such disulfide-linked complexes might reflect potential staggering of the complexes along the rim of incisures, because the width and pitch of our twisted ribbon model approximately fit in situ observations of incisure size and disc-stacking height, respectively (*19*). However, recent cryo-ET of mature incisures demonstrated a lack of zipper-like structure and no interconnection between membrane discs (*19*), although they were previously proposed (*15*, *35*). Thus, this study of detergent-solubilized complexes shows that PRPH2 and ROM1 by themselves may form large and intricate structures, which require further investigation by in situ cryo-ET to confirm the biological relevance.

In summary, the high-resolution PRPH2-ROM1 heterodimer structure and the observed oligomeric and multimeric arrangements of detergent-solubilized complexes, together with recently published in situ cryo-ET data of disc rims (*19*), indicate that PRPH2-ROM1 oligomerization may adopt varying arrangements and, hence, support the complex molecular processes of membrane remodeling in OS morphogenesis and stabilizing OS morphology (Fig. 4B). The high-resolution PRPH2-ROM1 heterodimer structure reveals the critical details of the minimal building unit and offers direct insight into individual pathogenic PRPH2 and ROM1 mutations, which could further affect future retinal disease–related therapeutic studies. The cryo-ET study of disc rims revealed a scaffold of three noncovalent dimers with a highly negative curvature, yielding the ca. 24-nm height of internalized disc rims (*19*). Scaffolds are stacked sideways, repetitively, linking dimers covalently through disulfide bonds with a very small negative curvature. The detergent-solubilized oligomers and multimers that we observed predominantly consist of multiple disulfide-linked dimers that display a positive curvature. In addition, we also observed occasionally negatively curved detergent-solubilized oligomers (Fig 4C). They possibly represent relaxed versions of the noncovalent protein scaffolds as observed by cryo-ET in mature disc rim (*19*), because they display a similar topology but with less curvature. Presumably, further protein interactions are present in mature discs that provide the tightness of the rims that are absent in the detergent-solubilized structure. Moreover, the infrequent occurrence of negatively curved structures among the detergent-solubilized particles is in line with the weak interactions maintaining these complexes. Overall, the observation of both positively and negatively curved oligomers fits with the multifaceted morphogenesis process that starts with cilium evagination and membrane outgrowth and ends in disc internalization (*5*) (Fig 4B). Moreover, the observation of a second micelle surrounding the C-terminal tails hints at a specific role for PRPH2 and ROM1 in the membrane remodeling process. Thus, the structures of PRPH2-ROM1 oligomers offer starting points for future studies toward deciphering the molecular basis of the multifaceted and dynamic membrane remodeling in photoreceptor morphogenesis.

## MATERIALS AND METHODS

### Constructs

Codon-optimized complementary DNA (cDNA) for mammalian cell expression, encoding for human PRPH2 and ROM1, was purchased from GeneArt. Full-length PRPH2 and ROM1 were cloned in a pUPE expression vector (U-Protein Express BV, the Netherlands) with either an N-terminal Strep-II tag, an N-terminal Strep_3_–green fluorescent protein (GFP) tag, or an N-terminal 6x-His tag, using 5′ Bam H1 and 3′ Not 1 restriction sites. PRPH2 and ROM1 mutant constructs were generated with polymerase chain reactions using the Q5 Site-Directed Mutagenesis Kit (New England Biolabs). All plasmids were confirmed by sequencing.

### Nanobody construct and purification

Nanobody19 was designed using the backbone of Nb8195 (*37*) and the hypervariable region sequences H1, H2, and H3 of Nb19 (*20*). Codon-optimized cDNA for *Escherichia coli* expression was purchased from GeneArt. Nanobody19 was cloned in a C-terminal 6x-His tag, using 5′ Bam H1 and 3′ Not 1 restriction sites and transformed into *E. coli* BL21(DE3). One single colony was precultured in 50 ml of LB supplemented with kanamycin (100 g/ml), 2% (w/v) glucose, and 1 mM MgCl_2_ at 170 rpm and 37°C overnight. A secondary culture of 1 liter of LB-kanamycin was inoculated with 10 ml of the preculture. This culture was grown at 37°C and 170 rpm until the optical density at 600 nm reached 0.7. Nanobody expression was induced by addition of 1 mM isopropyl-β-d-thiogalactopyranoside and grown at 170 rpm and 28°C overnight. Cells were harvested by centrifuging at 5000*g* for 20 min, resuspended in 15 ml of ice-cold *N*-tris(hydroxymethyl)methyl-2-aminoethanesulfonic acid (TES) buffer [200 mM tris-HCl (pH 8), 0.5 mM EDTA, and 0.5 M sucrose] and incubated, by shaking at 4°C for 1 hour. Thirty milliliters of fourfold diluted TES buffer was added to the resuspended pellet and incubated, by shaking at 4°C for 45 min. The suspension was centrifuged for 30 min at 10,000*g* at 4°C, the supernatant was recovered and incubated with Ni–nitrilotriacetic acid beads (Qiagen) for 1 hour at 4°C. Beads were washed using 15 mM imidazole, 25 mM tris-HCl, and 150 mM NaCl at pH 7.5, and Nanobody19 was eluted using 500 mM imidazole and injected onto a Superdex 200 Increase 10/300 GL (GE Healthcare Life Sciences) SEC column preequilibrated with 25 mM tris-HCl and 150 mM NaCl at pH 7.5. Fractions containing Nanobody19 were pooled and stored at −20°C for further use.

### Expression and purification of PRPH2-ROM1 heterodimer with Nanobody19

N-terminal 6x-His–tagged PRPH2^C150S^ and N-terminal Strep_3_-GFP–tagged ROM1 were diluted with dummy plasmid expressing the tripeptide methionine-glycine-serine (MGS) and transiently transfected into 2 liters of HEK293T cells (provided by U-Protein Express BV). Cells were grown at 37°C and harvested by centrifugation (10 min, 1000*g* at 4°C) after 96 hours. The harvested cell pellets were lysed and solubilized for 2 hours at 4°C with gentle agitation in buffer A comprising 50 mM tris-HCl, 150 mM NaCl at pH 7.5, EDTA-free complete protease inhibitor tablet (Roche), 1% (w/v) DDM, and 0.1% (w/v) CHS. Samples were ultracentrifuged (40 min, 40,000 rpm at 4°C), and supernatants were then incubated with Strep-Tactin Sepharose beads (GE Healthcare Life Sciences) for 2 hours at 4°C in a buffer containing 0.025% (w/v) DDM/CHS. Proteins were eluted in 2.5 mM *d*-desthiobiotin (Sigma-Aldrich) and injected onto a Superdex 200 Increase 10/300 GL (GE Healthcare Life Sciences) SEC column preequilibrated with buffer B comprising 25 mM tris-HCl, 150 mM NaCl at pH 7.5, and 0.025% (w/v) DDM/CHS. Fractions containing PRPH2-ROM1 heterodimer were pooled and incubated with an excess of Nanobody19 and concentrated in a 100-kDa concentration device (Amicon) to ~9 mg/ml.

### Expression and purification of PRPH2-ROM1 oligomers

N-terminal 6x-His–tagged PRPH2 and N-terminal Strep_3_-tagged ROM1 were diluted with dummy plasmid expressing the tripeptide MGS and transiently transfected into 2 liters of HEK293T cells (provided by U-Protein Express BV). Expression and purification were carried as for PRPH2-ROM1 heterodimer. One fraction containing heterogeneous PRPH2-ROM1 complexes was pooled and concentrated in a 100-kDa concentration device (Amicon) to ~3.6 mg/ml.

### Negative-stain EM

Fractions containing PRPH2-ROM1 complexes were diluted to 0.005 mg/ml in buffer B, and 3.5 μl of the sample was placed onto glow-discharged grid. After 20-s incubation, the sample was blotted with Whatman paper, dipped into 40 μl of distilled water, and stained with 20 μl of a filtered solution of 2% (w/v) uranyl acetate. Grids were imaged using a Tecnai 12 microscope (Field Electron and Ion Company, FEI) at 120 keV.

### Cryo-EM grid preparation and data collection

A total of 3.5 μl of PRPH2^C150S^-ROM1-Nb19 (9 mg/ml) or PRPH2-ROM1 (3.6 mg/ml) was pipetted into a glow-discharged R1.2/1.3 300 mesh Au holey carbon grids (Quantifoil) and plunge-frozen in liquid ethane/propane mixture or pure ethane with the help of a Vitrobot Mark IV (Thermo Fisher Scientific), blotting with force 0 for 4 s at 8°C with 90% humidity. Movies were collected on a 300-kV Titan Krios microscope (Thermo Fisher Scientific) equipped with a K3 summit detector (Gatan) and a postcolumn GIF Quantum energy filter operating in counted superresolution mode at a magnification of 130,000 giving a nominal pixel size of 0.328 Å. For the PRPH2-ROM1-Nb19 dataset, 10,448 movies were collected using a defocus range of −0.8 to −2.3 μm. A total dose of 60 e^−^/Å^2^ was reached using a dose rate of 19.1 e^−^/pixel/s per frame across 60 frames for 1.5-s exposure time. For PRPH2-ROM1 oligomers, two datasets were merged (8015 + 9100 movies) using similar defocus range and total dose.

### Cryo-EM data processing and 3D reconstruction

The cryo-EM data processing workflow is summarized in figs. S3 and S10. The map and model of the PRPH2-ROM1 heterodimer complex have been deposited in the Electron Microscopy Data Bank with ID EMD-14991 and in the Protein Data Bank (PDB) with ID 7ZW1. Maps of PRPH2-ROM1 oligomers have been deposited at EMBD, tetramer (ID: EMD-15021), hexamer (EMD-15023), and octamer (ID: EMD-15020). All processing steps were performed using cryoSPARC v3.2/3 (*38*).

#### Heterodimer (fig. S3)

A total of 10,448 cryo-EM movies were recorded. Patch-based motion correction and Patch-based contrast transform function (CTF) estimation were performed in cryoSPARC (v3.2/3) using the Patch Motion and Patch CTF jobs. After curations of the exposures, 9811 movies were merged, and a first round of Blob picker was performed on 50 movies. After extraction of the picked particles and 2D classification, 2D class templates were used, and the whole dataset was subjected to Template Picker job. Particles (2,796,694) were extracted in 700-pixel box size, and Fourier was cropped to 150 pixels for 2D classification. Multiple rounds of 2D classification were performed to isolate clean particles, yielding to 465,338 particles, which were reextracted in 700-pixel box for further 3D cleaning. One good ab initio model reconstruction was built after 2D selection of 394,318 particles, while the rest of the particles was subjected to two ab initio models to generate data-driven decoy models. Multiple rounds of heterogeneous refinements and nonuniform refinements were performed using one good initial model and two decoy models. This allowed isolation of a subset of 93,000 particles that resulted in a 4.0-Å map. Reextraction of the particle subset in 432-pixel box resulted in 3.66-Å resolution. The map was sharpened using DeepEMhancer (*39*), both half maps were used as input, and the high-resolution parameter was used for sharpening.

#### Oligomers (fig. S10)

A total of 8015 and 9100 cryo-EM movies were recorded during two microscope sessions. Patch-based motion correction and Patch-based CTF estimation were performed on the cryo-EM movies individually in cryoSPARC (v3.2/3) using the Patch Motion and Patch CTF jobs. After curations of the exposures, 7582 and 8562 movies were merged, and a first round of Blob picker was performed on 50 movies. After extraction of the picked particles and 2D classification, 2D class templates were used, and the whole dataset was subjected to Template Picker job. Particles (3,968,113) were extracted in 756-pixel box size, and Fourier was cropped to 150 pixels for 2D and 3D cleanup. Multiple rounds of 2D classification were performed to isolate clean subsets, yielding to 699,031 particles. Two ab initio models reconstruction were performed for each of the three subsets of particles, each displaying different oligomers features (fig. S10). Multiple rounds of heterogeneous refinements and nonuniform refinements were performed using good 3D initial models for the dimer, tetramer, hexamer, and octamer, in addition of three decoys models to further clean the selected particles. This allowed isolation of a tetramer subset of 58,000 particles which were reextracted in 470-pixel box and nonuniform refinement resulted in a reconstruction at 8.2 Å. The hexamer and octamer subsets were merged and reextracted in 700-pixel box. The use of all merged particles and respective 3D map in a nonuniform refinement job resulted in a reconstruction of the hexamer 7.6 Å, and after heterogeneous refinement between hexamer and octamer, the octamer subset of 133,000 particles resulted in a 7.6-Å resolution map.

### Model building and refinement

Human PRPH2 and ROM1 AlphaFold models (AF-P23942-F1 and AF-Q03395-F1) were used as initial models for building PRPH2-ROM1 heterodimer. Nanobody19 model was generated using AlphaFold (*25*). Initial models were docked into the sharpened map and fitted manually using UCSF ChimeraX (*40*). Each model was then placed in Coot (*41*), followed by several rounds of real-space refinement using Phenix (*42*, *43*). Refinement and validation were done using the nonsharpened map and are summarized in table S2.

### Figure preparation

All the figures were prepared using Adobe Illustrator. All figures containing cryo-EM density maps and protein structures were generated using UCSF ChimeraX (*40*).
