# Supplementary material for: Cryo-EM structures of peripherin-2 and ROM1 suggest multiple roles in photoreceptor membrane morphogenesis
Source: Sci Adv. 2022 Nov 9;8(45):eadd3677. doi: 10.1126/sciadv.add3677 (PMC9645710; doi:10.1126/sciadv.add3677)
Supplement: Supplementary file 1 — Figs. S1 to S13 Tables S1 and S2 [file sciadv.add3677_sm.pdf]

Science Advances
AAAS

Supplementary Materials for

Cryo-EM structures of peripherin-2 and ROM1 suggest multiple roles in photoreceptor membrane morphogenesis

Dounia El Mazouni and Piet Gros

Corresponding author: Piet Gros, p.gros@uu.nl

Sci. Adv. **8**, eadd3677 (2022)
DOI: 10.1126/sciadv.add3677

This PDF file includes:

Figs. S1 to S13
Tables S1 and S2

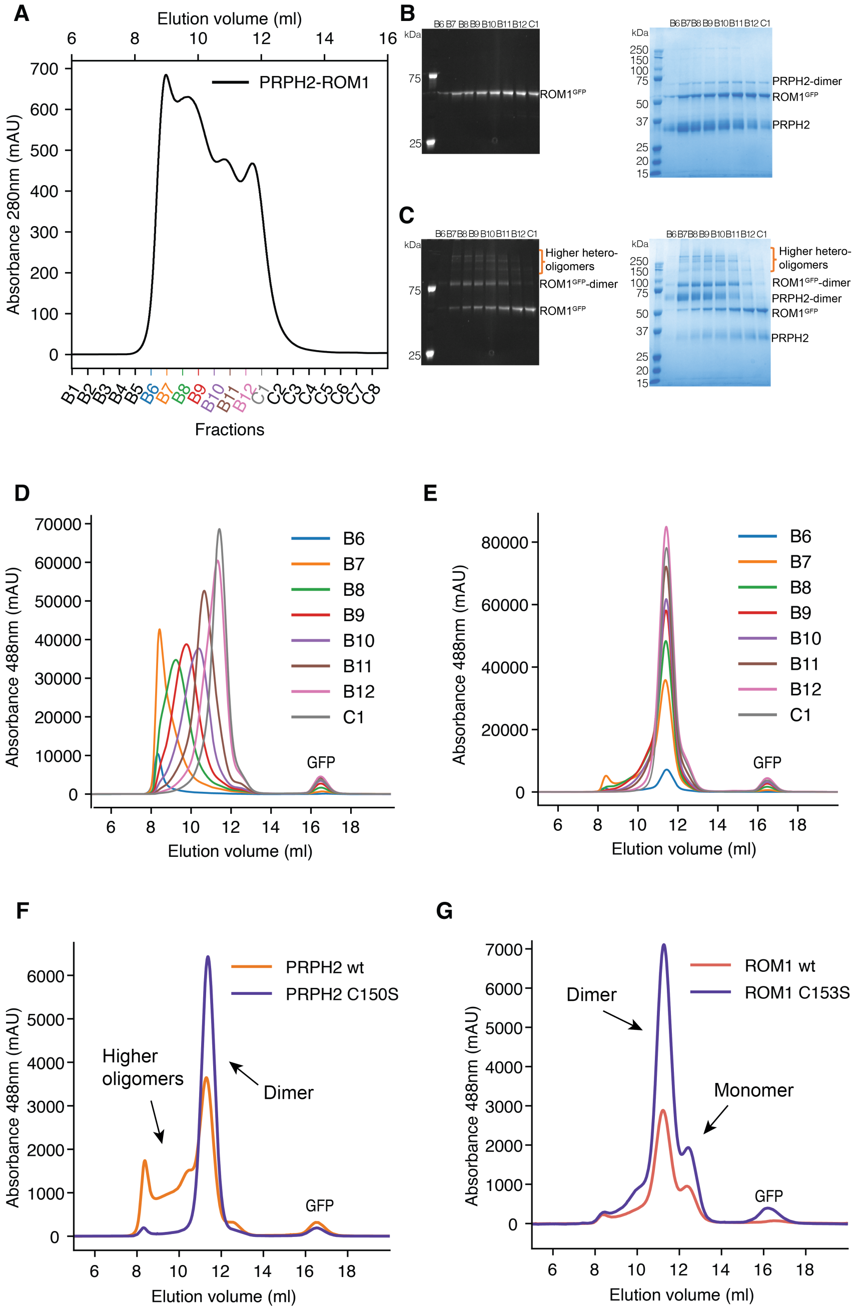

Fig. S1. Purification and biochemical characterization of human PRPH2-ROM1 complex, PRPH2 and ROM1. (**A**) Size-exclusion chromatography (SEC) elution profile of co-expressed PRPH2 -ROM1$^{STREP-GFP}$ in HEK-E+ cells after STREP affinity. (**B**) Reducing SDS-PAGE gel of elution peak fractions. (Left) GFP-fluorescent, (Right) Coomassie-stained. (**C**) Non-reducing SDS-PAGE gel of elution peak fractions. (Left) GFP-fluorescent, (Right) Coomassie-stained. (**D**) SEC profile of PRPH2-ROM1 elution fractions re-injected into SEC column connected to GFP fluorescence detector reveal overlapping peaks, indicating a range of oligomeric species**.** (**E**) SEC profile of PRPH2-ROM1 elution fractions with addition of DTT. (**F**) SEC elution profile of PRPH2 wt (orange) and PRPH2 C150S (blue). (**G**) SEC elution profile of ROM1 wt (red) and ROM1 C153S (blue).

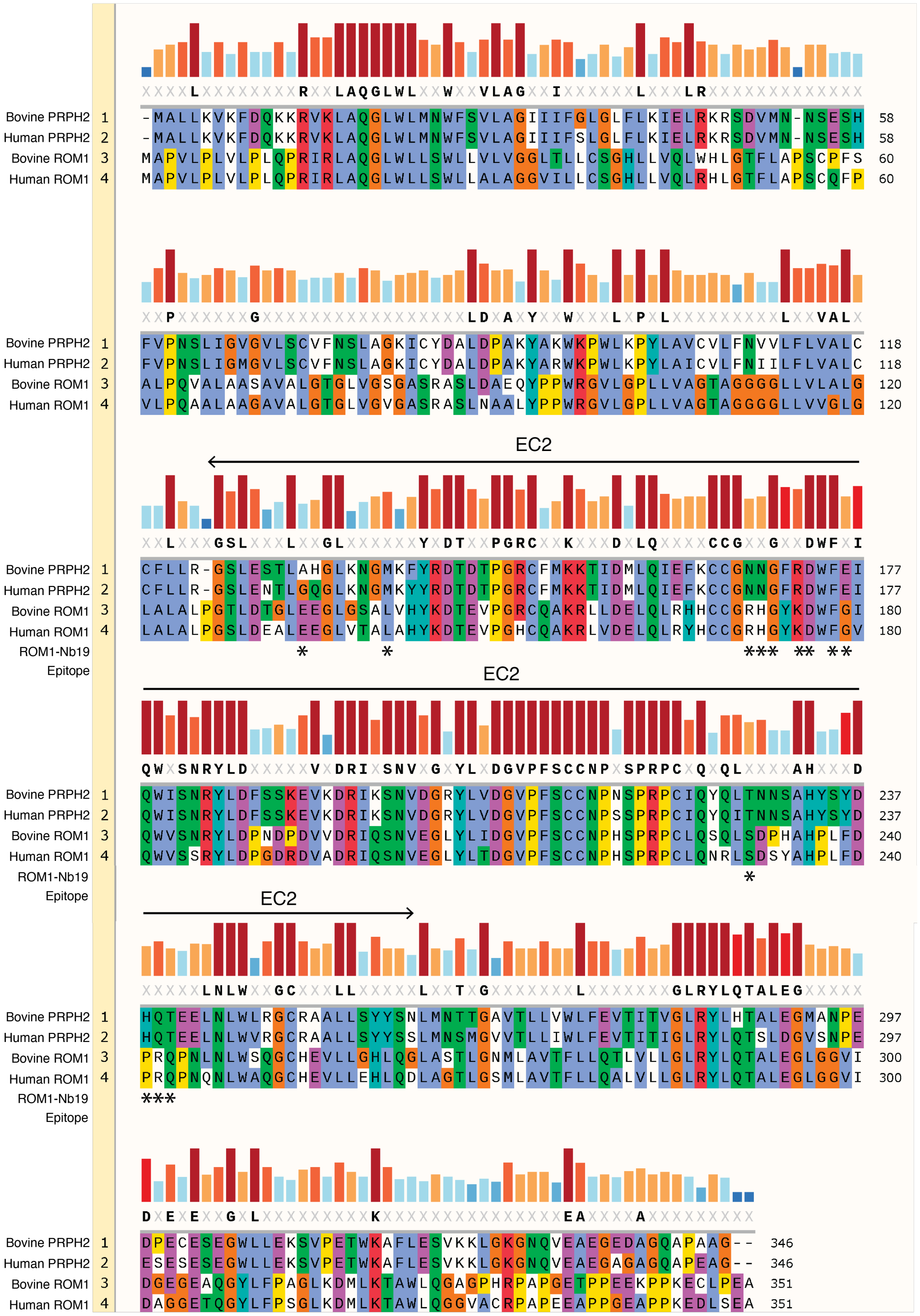

Fig S2. Sequence alignment between bovine and human PRPH2 and ROM1. EC2 of human PRPH2 and ROM1 is shown. Invariant and highly conserved residues are indicated above the sequences by blue and red bars, respectively, whereas less conserved to highly variable amino acids are indicated in light to dark blue bars. The character of amino-acid residues is coloured: hydrophobic (blue), positively charged (red), negatively charged (purple), in green when polar (green), glycine (orange), prolines (yellow) and finally aromatics (cyan). ROM1-Nb19 epitope is indicated with black stars.

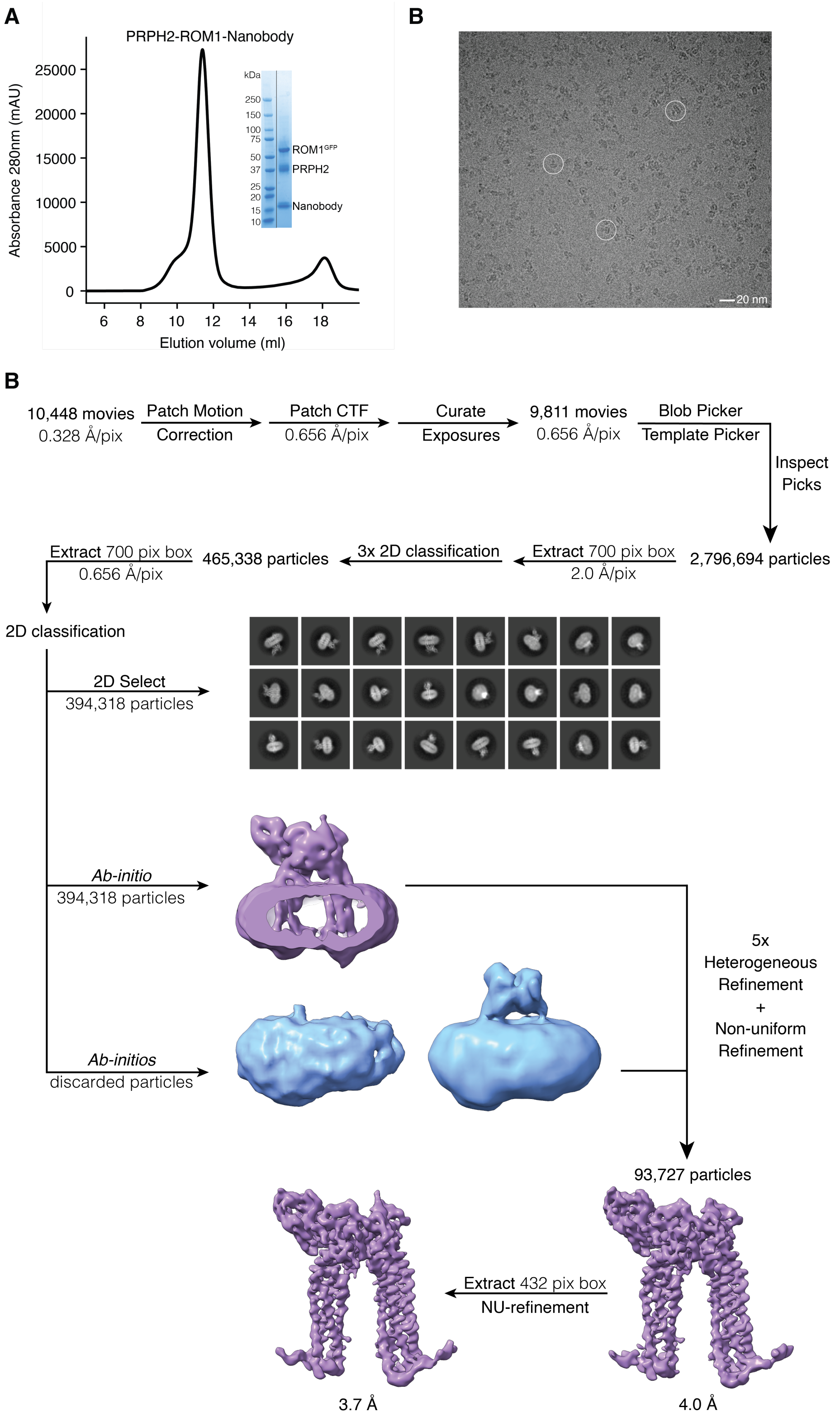

Fig. S3. Single-particle cryo-EM processing workflow of PRPH2-ROM1 dimer (**A**) Size-exclusion chromatography (SEC) profile of PRPH2-ROM1 EM sample. (**B**) Representative cryo-EM micrograph. Few particles are highlighted using a white circle. (**C**) Summary of cryo-EM processing pipeline used to obtain the final density map used for model building using cryoSPARC *(38)*.

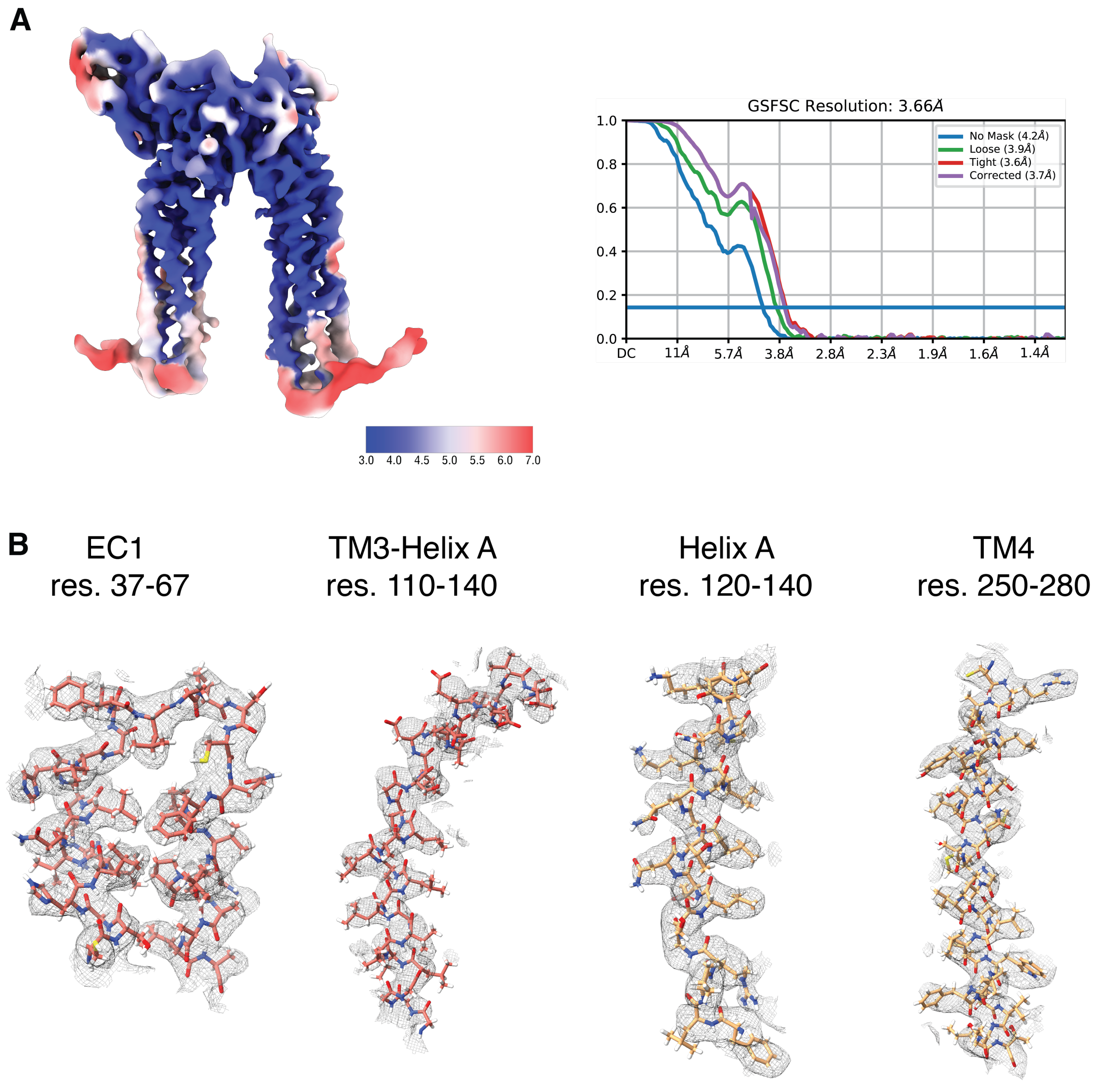

Fig. S4. Cryo-EM map quality for PRPH2-ROM1 dimer. (**A**) Cryo-EM map colored according to local resolution, calculated in cryoSPARC and Fourier shell correlation (FSC) curves. (**B**) Cryo-EM densities (transparent grey surface) are shown with corresponding segments of the atomic model; in stick representation EC1 and TM3-Helix A sidechains are shown for ROM1 and Helix A and TM4 for PRPH2.

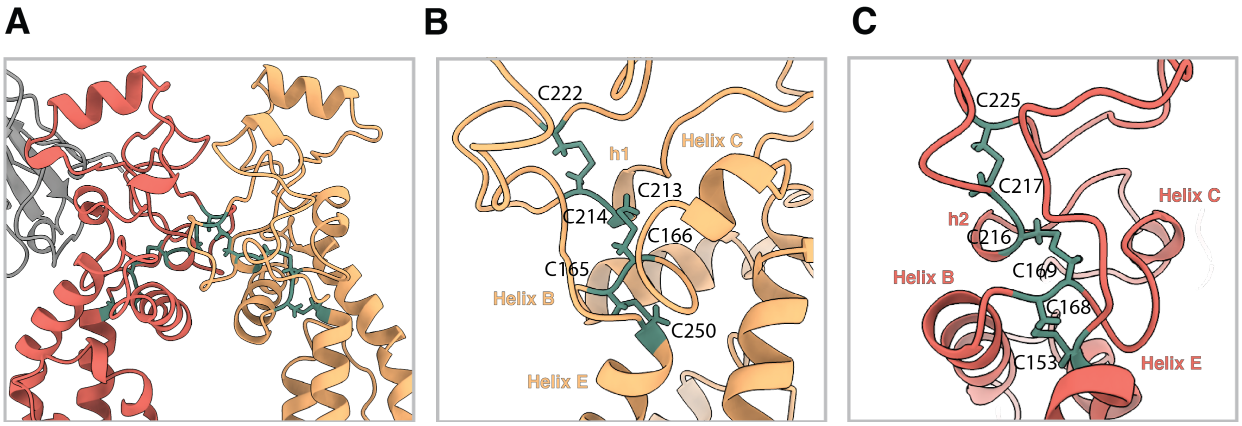

Fig. S5. Disulfide bonds stabilize the EC2 regions. (**A**) Zoom in PRPH2-ROM1 dimer interface with 3 disulfide bonds in each respective EC2s shown in green. (**B**) Zoom in PRPH2 EC2, the 3 disulfide bonds, Cys217-Cys225, Cys169-Cys216 and Cys168-Cys253 are shown in green. (**C**) Zoom in ROM1 EC2, the 3 disulfide bonds, Cys165-Cys250, Cys166-Cys213 and Cys214-Cys222 are shown in green.

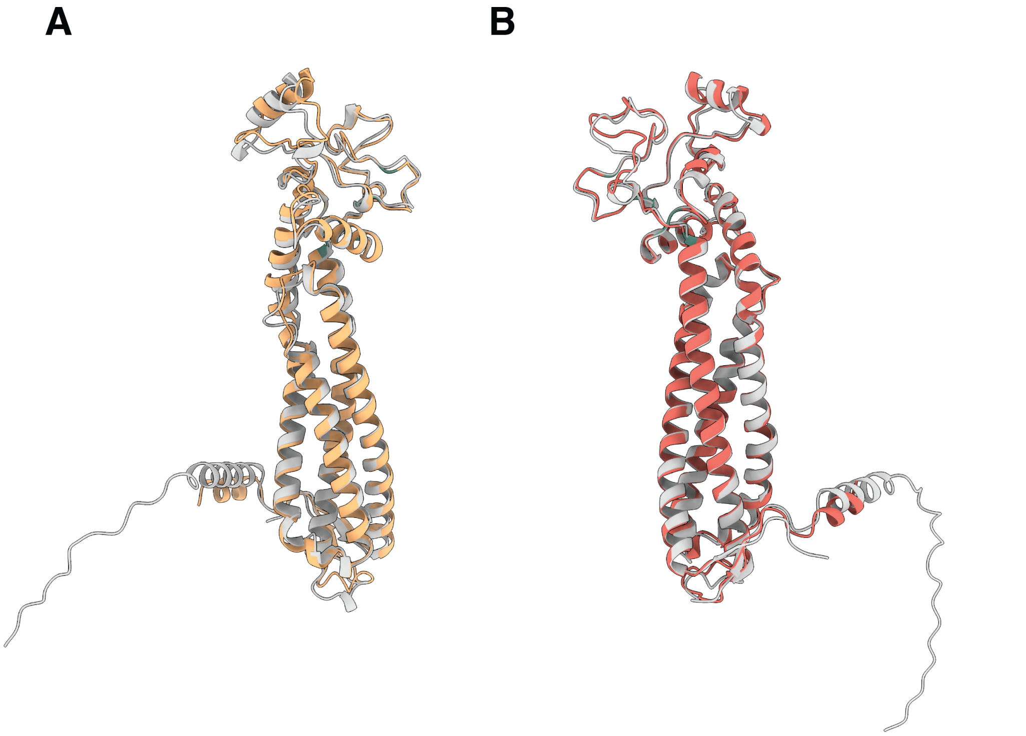

Fig. S6. Comparison of experimental PRPH2 and ROM1 structure with predicted Alphafold models. (**A**) Experimental PRPH2 structure in orange, predicted model in grey. (**B**) Experimental ROM1 structure in red, predicted model in grey.

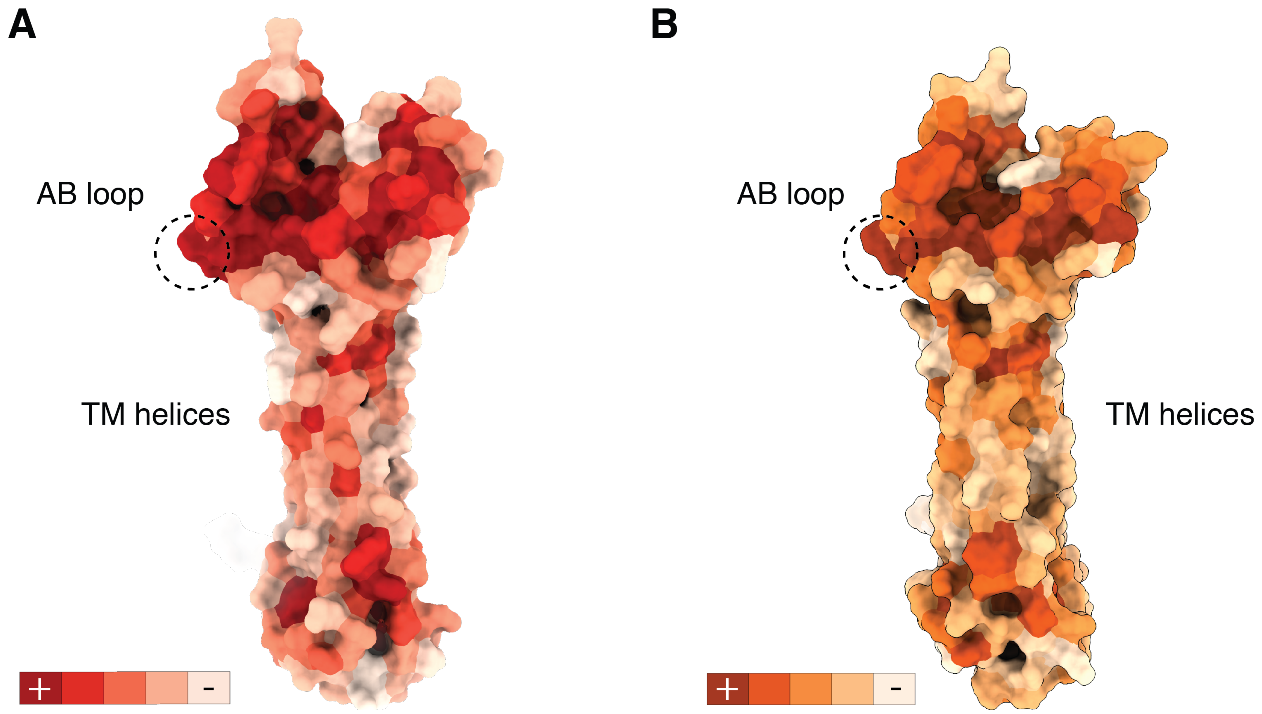

Fig. S7. PRPH2-ROM1 sequence conservation. (**A**) Open book surface representation of ROM1 colored according to the sequence conservation from the most conserved (dark red) to the most divergent (light red), using the ConSurf *(24)* server based on an alignment of 100 homologues across species. (**B**) Open book surface representation of PRPH2 colored according to the sequence conservation from the most conserved (dark orange) to the most divergent (light orange), using the ConSurf server based on an alignment of 100 homologues across species.

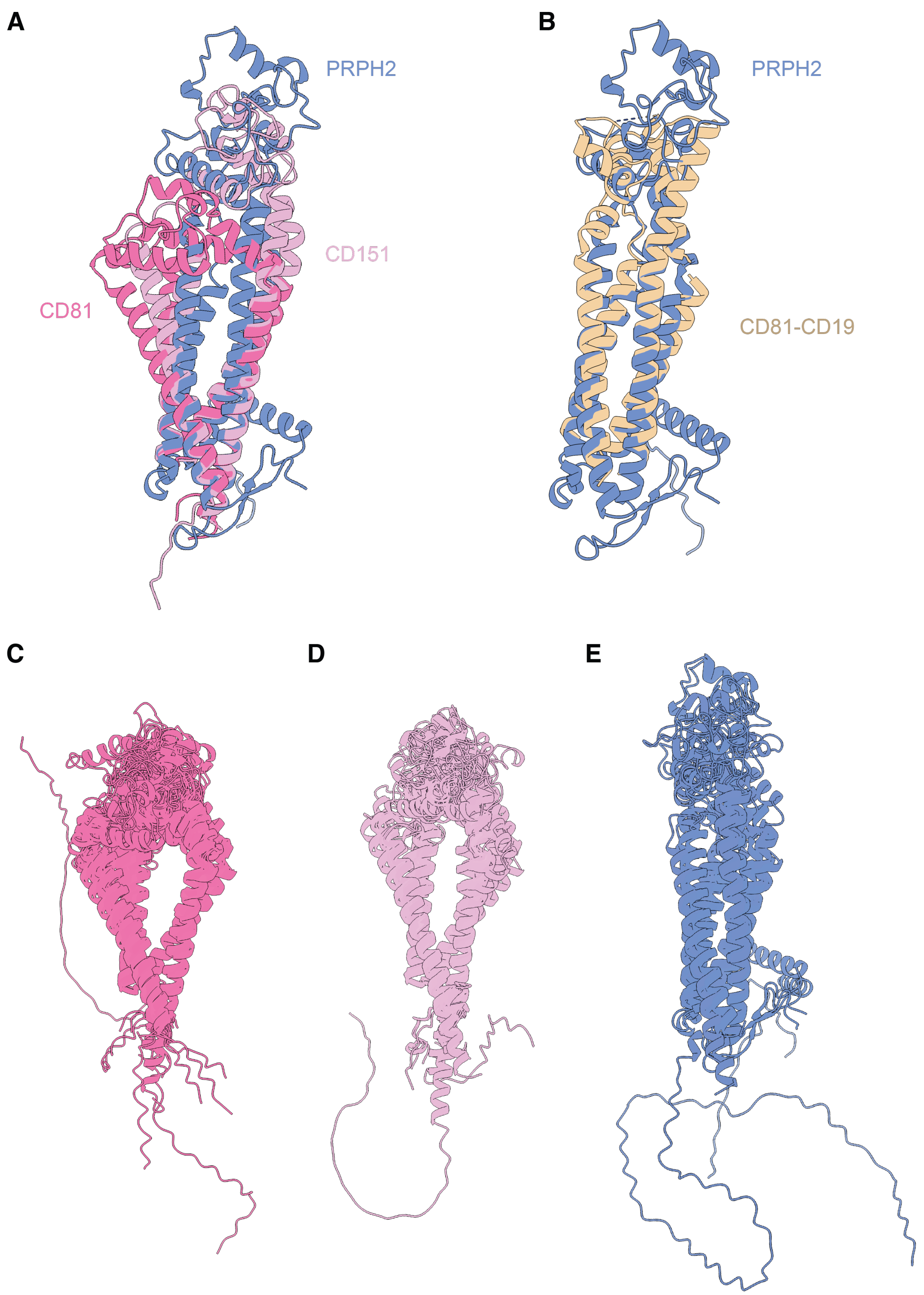

Fig. S8. Structural comparison with tetraspanin family. (**A**) Superimposition of alphafold-structure predictions of CD81, CD151 and PRPH2. (**B**) Superimposition of PRPH2 and CD81 in CD81-CD19 structure. (**C**) Superimposition of human tetraspanins in wide cone shape (TSPAN1, TSPAN2, TSPAN9, CD9, CD82, TSPAN5, TSPAN14, TSPAN10, TSPAN16, TSPAN33, TSPAN17, TSPAN18, CD37, CD53, CD81). (**D**) Superimposition of human tetraspanins in narrow cone shape (TSPAN11, TSPAN4, TSPAN6, TSPAN3, TSPAN13, TSPAN15, TSPAN12, TSPAN19, CD63, TSPAN7, CD151, TSPAN31). (**E**) Superimposition of human tetraspanins with a cylindrical shape (TSPAN21, TSPAN20, TSPAN32, PRPH2, ROM1).

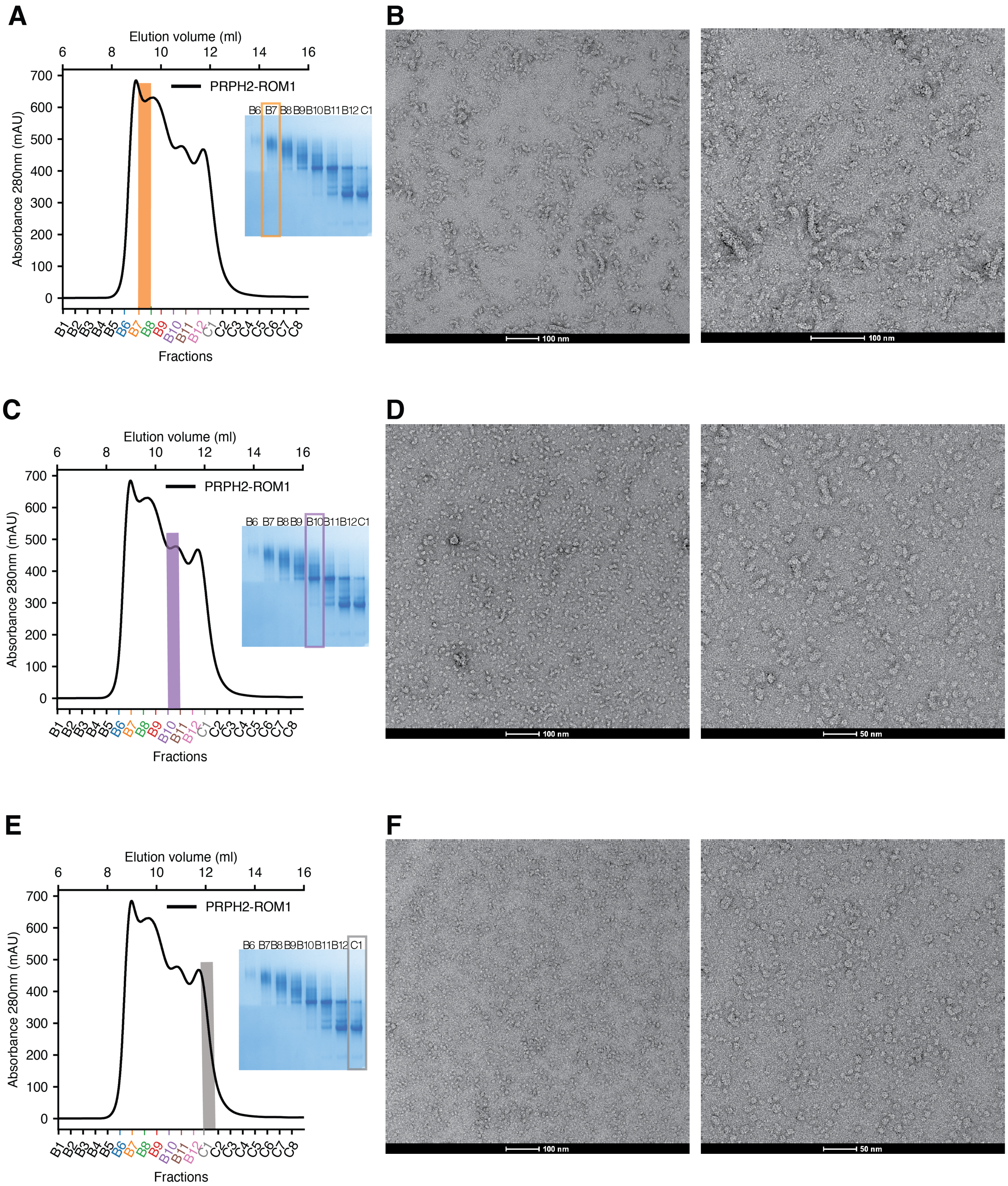

Fig. S9. Negative-stain micrographs of purified PRPH2-ROM1. (**A**) SEC elution profile of co-expressed PRPH2-ROM1$^{STREP\text{-}GFP}$ and relative native–PAGE gel. Fraction B7 is highlighted in orange. (**B**) Representative negative-stain micrographs of fraction B7 exhibit heterogeneous and long particles. (**C**) Fraction B10 is highlighted in violet. (**D**) Representative negative-stain micrographs of fraction B10 exhibit few particles species. (**E**) Fraction C1 is highlighted in grey. (**F**) Representative negative-stain micrographs of fraction C1 exhibit homogeneous particles.

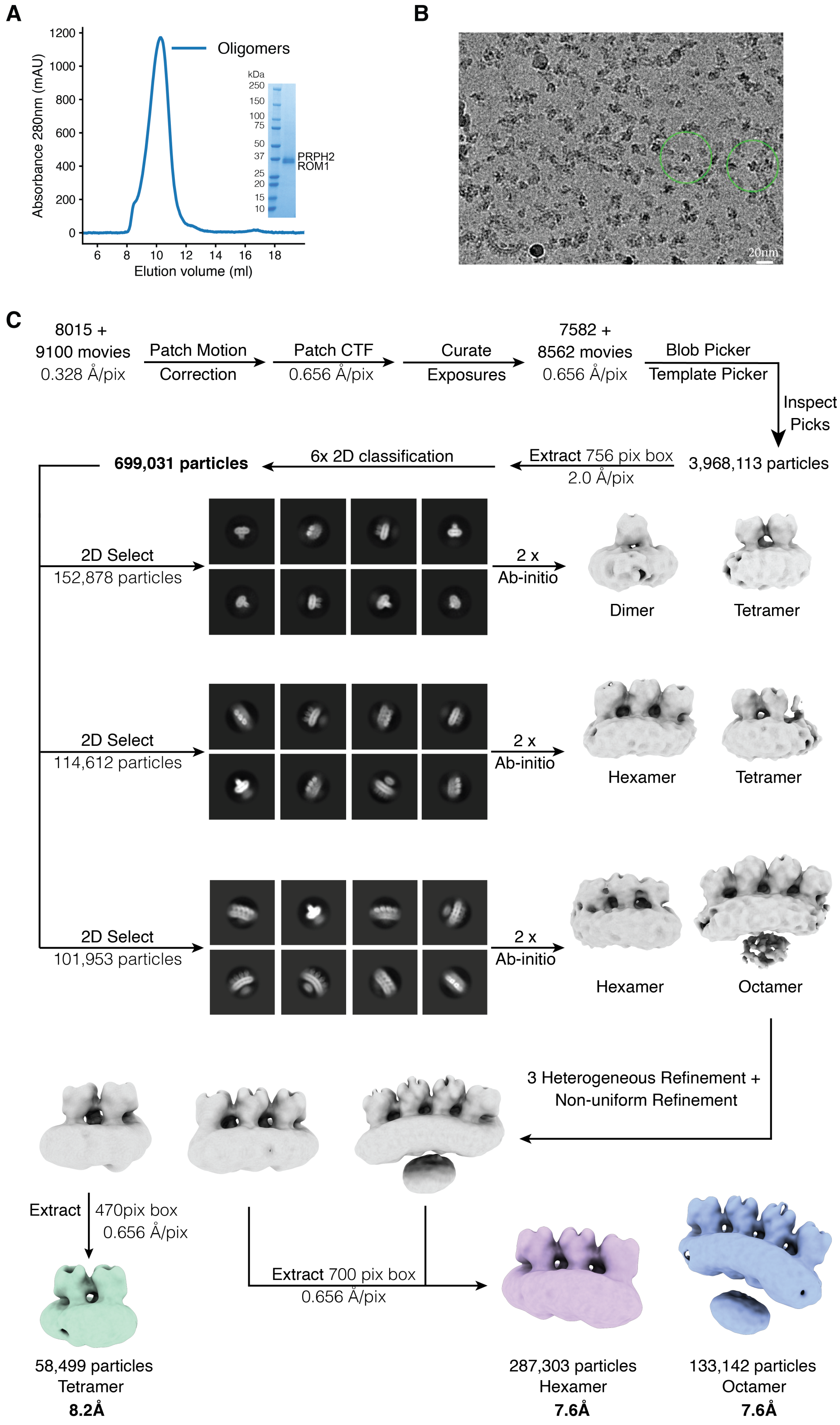

Fig. S10. Single-particle cryo-EM processing workflow of PRPH2-ROM1 oligomers (**A**) Size-exclusion chromatography (SEC) profile of PRPH2-ROM1 EM sample. (**B**) Representative cryo-EM micrograph. Two particles are highlighted using a green circle. (**C**) Summary of cryo-EM processing pipeline used to obtain the final density map using cryoSPARC.

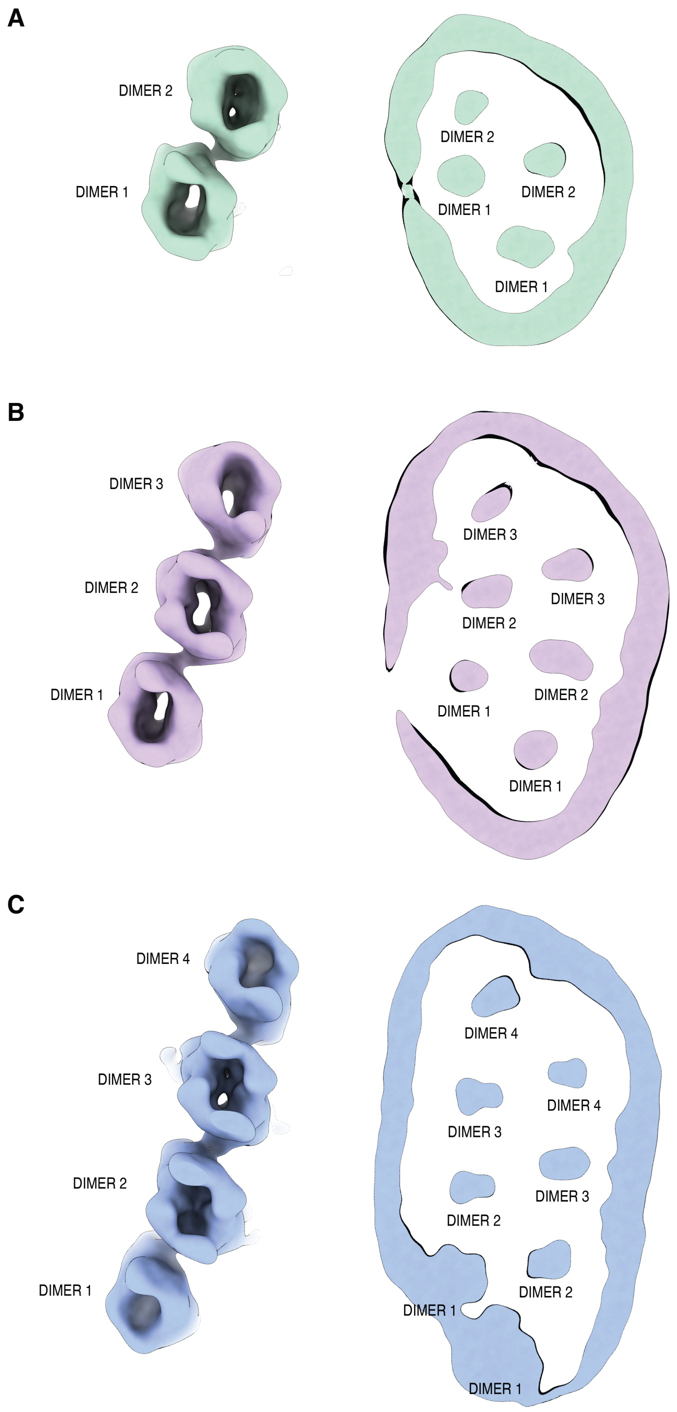

Fig S11. PRPH2-ROM1 staggered alignment. Cryo-EM maps with top view revealing intermolecular disulfide bridges and slice through the PRPH2-ROM1 detergent micelle revealing transmembrane densities of (**A**) tetramer, (**B**) hexamer, (**C**) octamer.

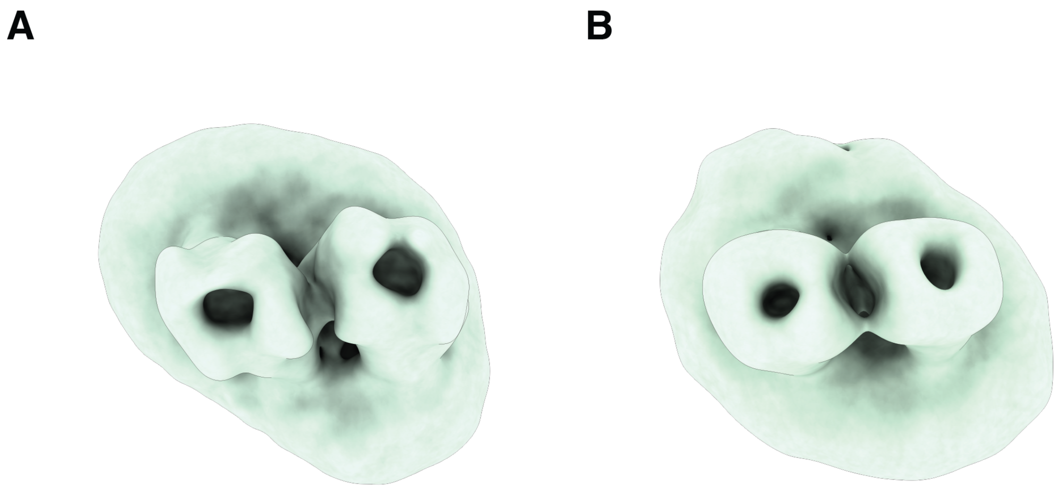

Fig S12. Alternative tetramer model. (A) 3D models of PRPH2-ROM1 tetramers with two disulphide-liked dimers (**B)** Alternative 3D form of PRPH2-ROM1 tetramers with a contact interface between the EC2 heads.

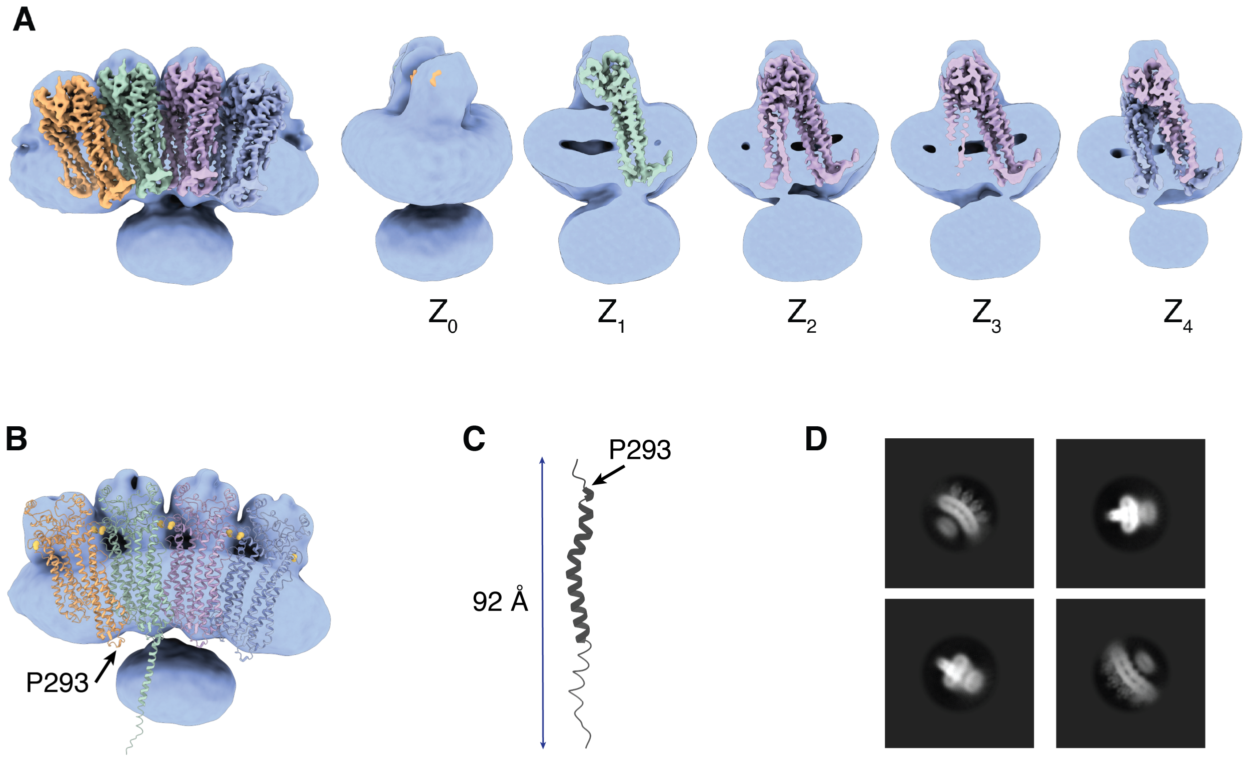

Fig. S13. C-terminal amphipathic helices undergo conformation change. (A) Cryo-EM map of PRPH2-ROM1 octamer with high-resolution dimers fitted into corresponding density transparently visible, front view and side view shown with side view correspond to Z_0. **(B)** PRPH2-ROM1 octamer atomic model lacking C-terminal domains and prediction PRPH2 C-terminal residues 296-246 attached to dimer 2. **(C)** Alphafold model prediction of PRPH2 C-terminal residues 296-346. **(D)** 2D classes with front and side view of PRPH2-ROM1 octamers showing extra-density.

Table S1. PRPH2 and ROM1 disease-causing mutations. Database HGMD *(23)*

PRPH2 Cytoplasmic (21 mutations)			PRPH2 Transmembrane (15 mutations)			PRPH2 EC1 (3 mutations)		
Residues	Mutations	Probability of deleterious mutation	Residues	Mutations	Probability of deleterious mutation	Residues	Mutations	Probability of deleterious mutation
1	Met-Leu		25	Trp-Cys		45	Leu-Phe	0.72; High risk
1	Met-Thr	0.79; High risk	27	Ser-Phe	0.64; High risk	46	Arg-Term	
13	Arg-Gln		32	Ile-Val	0.58; Medium risk	50	Asp-His	
13	Arg-Trp		39	Leu-Pro				
15	Lys-Arg		68	Gly-Arg	0.98; Very high risk			
21	Trp-Term		101	Tyr-Term				
82	Cys-Term		116	Ala-Ser				
91	Tyr-His		123	Arg-Trp	0.84; Very high risk			
91	Tyr-Asn		266	Gly-Asp	0.99; Very high risk			
94	Trp-Term		268	Val-Ile	0.34; Low risk			
305	Gly-Asp	0.69; High risk	276	Glu-Term				
309	Glu-Asp		285	Tyr-Term				
313	Pro-Leu		286	Leu-Pro				
313	Pro-Ser		288	Thr-Lys				
316	Trp-Term		289	Ser-Leu	0.36; Low risk			
316	Trp-Gly	0.43; Medium risk						
321	Glu-Term							
331	Gln-Term							
332	Val-Glu							
337	Ala-Thr							
338	Asp-Gly							

PRPH2 EC2 (127 mutations)

Residues	Mutations	Probability of deleterious mutation	Residues	Mutations	Probability of deleterious mutation	Residues	Mutations	Probability of deleterious mutation
125	Ser-Leu	0.47; Medium risk	184	Tyr-Ser	0.82; Very high risk	215	Asn-Tyr	
126	Leu-Pro	0.95; Very high risk	185	Leu-Pro	0.81; Very high risk	216	Pro-Leu	0.85; Very high risk
126	Leu-Arg	0.93; Very high risk	186	Asp-Asn		216	Pro-Ser	0.81; Very high risk
127	Glu-Gly		191	Glu-Term		216	Pro-Arg	
130	Leu-Pro		194	Asp-Glu		217	Ser-Gly	
135	Lys-Glu		195	Arg-Term		218	Ser-Pro	
137	Gly-Asp	0.68; High risk	195	Arg-Gly		218	Ser-Term	
141	Tyr-His	0.89; Very high risk	195	Arg-Gln		219	Pro-Arg	0.87; Very high risk
141	Tyr-Cys	0.94; Very high risk	195	Arg-Leu	0.95; Very high risk	220	Arg-Pro	
142	Arg-Gln		196	Ile-Asn		220	Arg-Gln	0.95; Very high risk
142	Arg-Trp	0.61; High risk	196	Ile-Phe		220	Arg-Trp	0.88; Very high risk
152	Met-Val	0.34; Low risk	197	Lys-Glu	0.69; High risk	221	Pro-Leu	0.93; Very high risk
153	Lys-Glu		198	Ser-Arg		222	Cys-Arg	
153	Lys-Thr		199	Asn-Asp		222	Cys-Ser	
153	Lys-Arg	0.8; Very high risk	200	Val-Gly		222	Cys-Tyr	
154	Lys-Gln		200	Val-Glu	0.89; Very high risk	222	Cys-Ser	
155	Thr-Ile		202	Gly-Glu		223	Ile-Asn	
157	Asp-Asn	0.94; Very high risk	204	Tyr-Term		225	Tyr-Term	
160	Gln-Term		204	Tyr-His		226	Gln-Term	
161	Ile-Met		204	Tyr-Term		228	Thr-Ile	

165	Cys-Phe		205	Leu-Pro		229	Arg-His	
165	Cys-Tyr	0.97; Very high risk	207	Asp-Gly		231	Ser-Term	
165	Cys-Arg	0.97; Very high risk	208	Gly-Asp	0.68; High risk	232	Ala-Pro	
166	Cys-Tyr		209	Val-Phe		236	Tyr-Term	
167	Gly-Ser	0.99; Very high risk	209	Val-Ile	0.76; High risk	239	Gln-Term	
167	Gly-Asp	0.98; Very high risk	209	Val-Asp		242	Glu-Gly	
170	Gly-Ser	0.7; High risk	210	Pro-Arg	0.95; Very high risk	244	Asn-Lys	
171	Phe-Cys		210	Pro-Leu	0.98; Very high risk	244	Asn-His	0.91; Very high risk
172	Arg-Gly	0.69; High risk	210	Pro-Ser	0.96; Very high risk	244	Asn-Lys	0.96; Very high risk
172	Arg-Trp	0.7; High risk	211	Phe-Leu		246	Trp-Cys	
172	Arg-Gln	0.59; Medium risk	211	Phe-Leu	0.94; Very high risk	246	Trp-Arg	0.78; High risk
173	Asp-Ala		212	Ser-Gly	0.93; Very high risk	249	Gly-Ser	0.97; Very high risk
173	Asp-Val	0.95; Very high risk	212	Ser-Thr	0.88; Very high risk	250	Cys-Arg	
174	Trp-Ser		213	Cys-Phe	0.96; Very high risk	250	Cys-Ser	
174	Trp-Cys		213	Cys-Ser		250	Cys-Gly	
174	Trp-Term		213	Cys-Trp		250	Cys-Tyr	
178	Gln-Arg	0.91; Very high risk	213	Cys-Arg	0.99; Very high risk	250	Cys-Phe	0.89; Very high risk
179	Trp-Cys		213	Cys-Tyr	0.97; Very high risk	253	Ala-Asp	
179	Trp-Gly		214	Cys-Term		254	Leu-Gln	
179	Trp-Leu		214	Cys-Tyr	0.85; Very high risk	257	Tyr-Term	
179	Trp-Arg	0.97; Very high risk	214	Cys-Ser	0.95; Very high risk	258	Tyr-Term	
181	Ser-Cys		215	Asn-His		258	Tyr-Term	
						265	Met-Thr	

ROM1 Cytoplasmic (2 mutations)			ROM1 Transmembrane (5 mutations)			ROM1 EC1 (1 mutations)		
Residues	Mutations	Probability of deleterious mutation	Residues	Mutations	Probability of deleterious mutation	Residues	Mutations	Probability of deleterious mutation
85	Arg-Trp		39	Ser-Arg		60	Pro-Thr	
94	Trp-Term		43	Leu-Arg				
			75	Gly-Asp	0.71; High risk			
			108	Thr-Met				
			113	Gly-Glu	0.84; Very high risk			
ROM1 EC2 (7 mutations)								
Residues	Mutations	Probability of deleterious mutation						
223	Arg-Gln							
224	Pro-Leu							
229	Arg-His							
238	Leu-Pro							
242	Arg-Gln	0.35; Low risk						
242	Arg-Term							
253	Cys-Tyr							

Table S2. Cryo-EM data collection and validation statistics

**PRPH2-ROM1**	**Heterodimer** EMD-14991 PDB 7ZW1	**Tetramer** EMD-15021	**Hexamer** EMD-15023	**Octamer** EMD-15020
**Data collection and processing**				
Magnification	130,000	130,000	130,000	130,000
Voltage (kV)	300	300	300	300
Electron exposure (e-/ Å^2)	60	60	60	60
Defocus range (μm)	0.8-2.3	0.8-2.3	0.8-2.3	0.8-2.3
Pixel size (Å)	0.656	0.656	0.656	0.656
Symmetry imposed	C1	C1	C1	C1
Initial micrographs	10,448	17,115	17,115	17,115
Initial particles	2,796,694	3,968,113	3,968,113	3,968,113
Final particles	93,727	58,499	287,303	133,142
Map resolution (Å)	3,7	8,2	7,6	7,6
**Refinement**				
Nonhydrogen atoms	11,807			
Protein residues	760			
Ligands	0			
Rms deviations				
Bond lengths (Å)	0.002			
Bond angles (°)	0.496			
Validation				
MolProbity score	1.6			
Clash score	7			
Poor rotamers (%)	0.32			
Ramachandran plot				
Favored (%)	96.95			
Allowed (%)	3.05			
Outliers (%)	0			
